# Antifungal Susceptibility Assessment of Innovative and Non-Conventional Lime Mortars Incorporating Almond-Shell Powder Bio-Waste Subjected to Particle-Dispersion Technique

**DOI:** 10.3390/ma17061426

**Published:** 2024-03-20

**Authors:** Alexandre Jerónimo, Mafalda Loureiro, Mariana Fernandes, Verónica De Zea Bermudez, Ana Briga-Sá

**Affiliations:** 1 CQ-VR, University of Trás-os-Montes e Alto Douro, 5000-801 Vila Real, Portugal; ajaj@utad.pt; 2 School of Science and Technology (ECT), University of Trás-os-Montes e Alto Douro, 5000-801 Vila Real, Portugal; mafaldalour@hotmail.com; 3 CQ-VR, Department of Chemistry, University of Trás-os-Montes e Alto Douro, 5000-801 Vila Real, Portugal; mspf@utad.pt (M.F.); vbermude@utad.pt (V.D.Z.B.); 4 CQ-VR, School of Science and Technology (ECT), University of Trás-os-Montes e Alto Douro, 5000-801 Vila Real, Portugal

**Keywords:** lime mortars, almond-shell powder, particle-dispersion techniques, fungal susceptibility, wettability, mechanical properties

## Abstract

A favorable environment for fungi colonization in building materials’ surfaces can emerge when certain hygrothermal conditions occur. Thus, reducing fungal growth susceptibility is of major interest. Furthermore, if the integration of bio-wastes is performed in parallel with the development of innovative materials for this purpose, a more sustainable and environmentally friendly material can be obtained. In this study, the fungal susceptibility of lime mortars incorporating almond-shell powder (*ASP*) microparticles (2 and 4%, wt.–wt. in relation to the binder content) was evaluated. The particle-dispersion technique was employed to prepare the bio-waste introduced in the mixtures. The fungal susceptibility of *ASP* samples was compared with nanotitania (n-TiO_2_) with recognized antifungal properties. Mechanical strength, water absorption, and wettability tests were also performed for a better characterization of the composites. Although the addition of 2% *ASP* led to mechanical properties reduction, an increase in the compressive and flexural strength resulted for 4% of the *ASP* content. Difficulties in fungal growth were observed for the samples incorporating *ASP*. No fungal development was detected in the mortar with 2% of *ASP*, which may be correlated with an increase in the surface hydrophobic behavior. Furthermore, mortars with *ASP* revealed a reduction in water absorption by capillarity ability, especially with 4% content, suggesting changes in the microstructure and pore characteristics. The results also demonstrated that an improvement in the physical and mechanical properties of the lime mortars can be achieved when *ASP* microparticles are previously subjected to dispersion techniques.

## 1. Introduction

Climate change and environmental deterioration are global threats requiring urgent action. In this context, several strategies emerged worldwide to mitigate the harmful impact on the environment. In Europe, the main goal of the European Green Deal is to transform the European Union into a modern, resource-efficient, and competitive economy to address these issues [1], ensuring there will be no greenhouse-gas emissions by 2050. These challenges cover different sectors of activity, namely the construction industry, aiming for more sustainable, efficient, and healthy solutions. However, innovative strategies for a greener building sector go far beyond architectural design. Construction organizations are now exploring ways to extract quality raw materials from what could previously have been considered waste, contributing to saving on costs and reducing the environmental impact [2]. 

In this framework, the nanotechnology sector is improving the green reputation of the building industry by reevaluating the raw materials required for construction and proposing the application of alternative techniques. The outstanding properties of carbon nanotubes, for instance, have already been explored for strength-enhancement and crack-prevention purposes in cement and concrete [3] and also for mechanical and thermal properties improvement in the case of ceramics [2]. In this context, nanotitanium dioxide (n-TiO_2_) is a particularly attractive material given its rich physical–chemical properties, biocompatibility, and wide variety of morphologies. TiO_2_ nanoparticles (NPs) are widely used in the construction industry because they impart self-cleaning properties to concrete. Furthermore, TiO_2_ NPs have photocatalytic properties that can be activated by ultraviolet radiation, giving rise to reactive oxygen species, which are very effective against fungi, since they attack the cell membrane and eventually cause them to be destroyed [4]. This attribute of TiO_2_ NPs is particularly attractive for building structures since these should not constitute a risk to human health, especially as far as hospital buildings are concerned.

In fact, when sufficient water exists in building materials, a suitable environment for fungi colonization is created. The presence of pathogenic microorganisms is known to cause serious health and environmental problems. Fungi need oxygen, water, carbon, nitrogen, and other micronutrients to grow. The combination of high levels of air moisture and high temperatures increases the amount of water and the risk of condensation in building-material surfaces, favoring microbial growth [5]. In contrast, in environments with low air temperatures and appropriate air exchange rates, fungi take a longer time to grow and colonize the surfaces. Fungal attack is a concern in terms of materials’ durability and the maintenance of indoor health conditions, being recognized as major biodeteriogens of both modern and historical buildings and monuments. Microbial contamination not only slowly deteriorates such monuments, but also destroys the culture represented by these types of buildings [6]. Special attention is given to this issue regarding stone and mortar artworks, showing that microbial communities’ colonization can accelerate the deterioration processes of the artworks and pose several challenges to their conservation [7]. Thus, fungi can be considered the most common cause of biodegradation, weakening materials’ functional properties, such as tensile strength, cracking, and pitting, as well as loss of color or discoloration [8].

Pesticides and biocides are used to control pests, such as fungi. Several studies in the literature report the use of different agents to combat microorganisms, namely in surface coatings [9,10]. However, these are chemical products that can cause pollution and harm wildlife if released into the air or spilled on the land, surface, or groundwater. They can also be harmful to human health if not properly stored and used [11,12]. Furthermore, the formation of biofilms, a collection of one or more types of microorganisms, including bacteria, fungi, and protists, on the materials’ surface [13] not only limits their function but also contributes to increasing the number of antibiotic-resistant microorganisms due to the excessive use of antibiotics or biocides to prevent their growth [9]. 

The problem of biodeterioration is increasingly important, given the growing popularity of sustainable construction practices using materials of natural origin [8]. In this context, more investigation is required to develop innovative materials to accomplish these functional requirements but also consider the sustainability and circular economy criteria. 

With the production model that is currently used, where raw material is extracted, a product is produced, then consumed, and finally discarded, there is a large generation of waste and energy dissipation throughout the production process [14]. Furthermore, the world’s ecological footprint exceeds the planet’s biocapacity by 50%, and at present, the world population needs 1.5 planets to meet its needs [14]. In addition, circular economy proposes the reuse of materials in the production cycle, with the objective of minimizing their deposition in the environment, thus avoiding the generation of negative environmental impacts [14].

In this framework, it is intended with the present research to evaluate the possibility of using bio-waste for building material purposes. Almond-shell powder (*ASP*), a residue resulting from almond production, was incorporated into lime mortar composition in order to analyze its influence on the biological susceptibility of the composite. 

Almond cultivation can be found in many regions around the world. *Prunus Dulcis* (*Rosaceae* family) [15] is an almond species that has been cultivated in the Mediterranean region for many centuries [16,17,18]. In Portugal, the almond sector is on a path of marked expansion, both in terms of area and production. This is especially true in the Alentejo and Beira Baixa regions, but also in the northern region of Trás-os-Montes, where the conversion of existing orchards into new orchards including almond trees is nowadays a reality. Portugal is already the third-largest producer of almonds in Europe. Almond production increased from 8713 tons in 2016 to 37,900 tons in 2021, and it is expected to achieve 50,000 tons in 2030 [19]. In order to produce 1 kg of almonds without shells, 3 to 5 kg of almonds with shells are necessary. Therefore, the yield is rather low (around 20–30%). The almond shell is a residue that is usually discarded or incinerated. This procedure not only pollutes the environment but also wastes a large amount of resources. Thus, several applications have been considered to reuse this by-product, taking advantage of its diverse properties. Almond shell is very popular in the cosmetics industry, and it is also identified as a sustainable alternative to microplastics. Its fibrous consistency, light-brown color, and low weight make it a valuable by-product ready to be integrated into composite mixtures (like wood–plastic composites), activated carbon for filter media or personal care items, wood glues, and bio-based resins [20]. This material has a well-developed pore structure, and the area around the holes is dense. It is characterized by a high volume of holes with a wide diameter range, making it a lightweight material. It was also stated that the mechanical properties of composites prepared from almond shells are superior to those of most biomass nutshells. Almond shells contain functional groups and active chemical properties which can enhance the physical and mechanical properties of composites [21]. 

As the almond shell is a residue that is normally discarded, the possibility of being used as raw material for building purposes will be a benefit from the circular economy perspective. In addition, if its incorporation enhances the antifungal properties of the material, it will curb the use of biocides. 

In this study, different contents of *ASP* microparticles were incorporated into the mortar composition and were previously subjected to particle dispersion, a well-known technique in the nanotechnology field [22]. The use of microparticles is related to the fact that the smaller the particle size, the greater the surface area, and consequently, the better the particle activity. Furthermore, subjecting these fine particles to dispersion can lead to an improvement in the paste’s performance. Agglomerate dispersion is a type of deagglomeration. In both situations, the same stressful techniques and resources are used. Agglomerates often require lower stress intensities, which are occasionally applied by shearing flows. A high stress intensity can probably encourage re-agglomeration, which is undesirable from the perspective of dispersing efficiency [23]. The agglomerates formed, whether permanent or not, in addition to influencing the rheology of the suspensions, can interfere with the packaging and, consequently, the microstructure of the material. Thus, the agglomerates must be eliminated, always looking for dispersed suspensions that, in addition to presenting lower viscosity, allow the use of higher concentrations of solid in the process. In these suspensions, the particles are individualized, being little influenced by the action of gravity, allowing them to remain homogeneous and stable for a longer period of time. The use of dispersing agents becomes necessary to avoid the sedimentation of particles and the consequent segregation of phases, enabling the preparation of homogeneous suspensions with high solids concentration [23]. Thus, considering the advantages of this nanotechnology and its generalized utilization in this field, it would be of great interest to evaluate the impact of its application to produce new materials for building purposes.

Thus, given the attractive properties reported for almond shell, the possibility of incorporating its microparticle powder as a raw material for lime-mortar production was investigated. The biological susceptibility of the *ASP*-based composites was compared with that of analog samples incorporating n-TiO_2_, which was considered the reference material on account of its anti-fungal properties. Herein the mechanical behavior, water absorption, and wettability of lime mortars with different contents of *ASP* were also analyzed. A conventional mortar was also used for comparison.

## 2. Materials and Methods

In this section, the materials used to produce the hydraulic lime mortars incorporating *ASP* will be characterized. The preparation of the samples and the experimental procedure to evaluate the physical and mechanical properties, and the biological susceptibility, will also be described.

### 2.1. Materials

**Figure 1:**
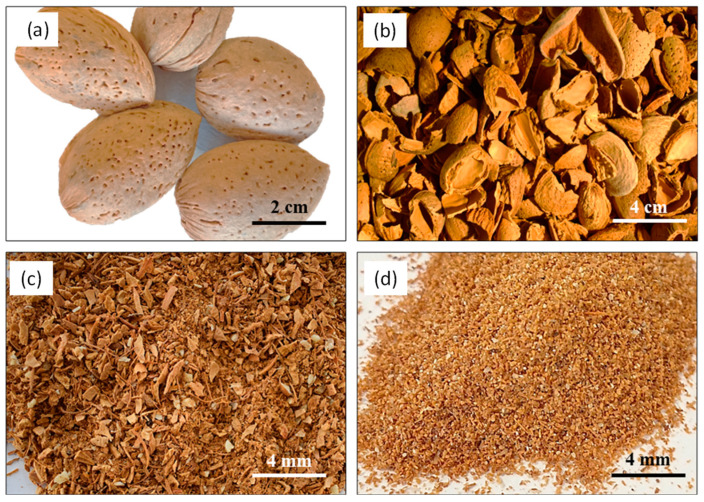
(**a**) Almond fruit, (**b**) raw almond shell, (**c**) crushed almond shell, and (**d**) *ASP*.

The *ASP* used in this research work was obtained from a company located northeast of Trás-os-Montes. The almond shell was crushed using a blender to obtain small particles, which were then sieved for the particle size distribution characterization. Figure 1a–d show the almond fruit, the raw almond shell, the crushed almond shell, and the *ASP* under study, respectively. The *ASP* presented a density value of 1058 kg/m^3^ and a particle size distribution < 250 µm. Natural hydraulic lime (NHL 5), with a density of 2700 kg/m^3^, was used as binder to produce the lime mortars under study. River sand, with a maximum particle size of 2 mm and a density of 2322 kg/m^3^, was incorporated as fine aggregate in the mixture’s composition. The n-TiO_2_ used in the reference mortar formulation was commercially available and presented a density value of 3800 kg/m^3^. A superplasticizer (*SP*) was added to improve the mixture’s workability, given the fineness of the *ASP*. When such fine particles were added to the mixture, more water was required to guarantee the workability and the plasticity of the mixture. So, adding *SP* to the mixture avoided more water addition and allowed the production of samples with higher workability. The *SP* incorporated into the lime mortar was polyacrylate, with a density value of 1050 kg/m^3^. Distilled water was employed in all the mixtures’ preparation.

### 2.2. Samples Preparation

**Figure 2:**
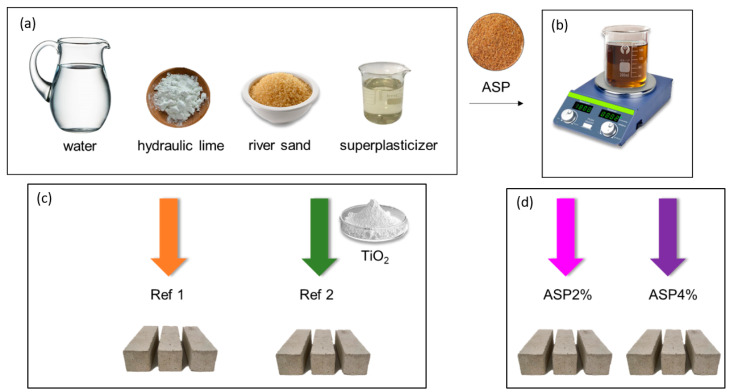
Preparation of the lime mortars: (**a**) components of the mortar’s mixture; (**b**) *ASP* particle dispersion; (**c**) reference mortars; (**d**) *ASP* mortars.

The preparation of the samples is schematically represented in Figure 2. Starting from the basic components (water, hydraulic lime, river sand, and *SP*), four different compositions were prepared. Two reference mixtures were produced: one without *ASP* (Ref1) as a control conventional mortar, and one without *ASP* but containing nTiO_2_ (Ref2) as a reference mortar with enhanced antifungal properties. For the other two, different contents (in %) of *ASP* were added to the mixture (*ASP*: NHL wt.–wt.): one with 2% of *ASP* (ASP2%) and, finally, one with 4% of *ASP* (ASP4%) (Table 1 and Figure 2). Previous studies reported that the introduction of fine particles in lime-mortar composites significantly increases the amount of water in the mixtures [24]. Thus, in this research work, it was decided to investigate the influence of *ASP* on the mortars’ properties when introduced in small amounts. The *ASP* content was calculated in relation to the amount of binder in the formulation, leading to 10 kg/m^3^ and 20 kg/m^3^ for percentages of 2% and 4%, respectively. 

The almond shell was ground and dispersed in water using the nanoparticle-dispersion technique [23], which involves suspensions of NPs in water or organic solvents [25], and then introduced into the mortar mixture. Subsequently, three prismatic specimens with dimensions of 16 cm × 4 cm × 4 cm were manufactured for each mixture for the flexural and compressive strength tests, and for the water absorption by capillarity evaluation. A circular sample of each of the mixtures was also prepared to be subjected to fungal activity. 

The specimens were cured in the laboratory at 20 °C and hermetically sealed for up to 28 days. 

### 2.3. Compressive and Flexural Strength Tests

An experimental procedure to assess the flexural and compressive strength of the mixtures was performed according to EN 1015-11 [26]. Three specimens were considered for each mortar composition to perform the flexural tests and six were used for the compression ones. 

The flexural strength was determined by three-point loading of the prismatic specimens to failure at 28 days of curing. The compressive strength of the mortars was then determined using the two pieces resulting from the previous experiment. Both mechanical tests were performed with a speed of 1 mm/s. The flexural strength (*f*), in N/mm^2^, was calculated by applying Equation (1) for each sample,

(1) $$f=1.5\times \frac{F\times l}{b\times d^{2}}$$

where *F* is the maximum load applied to the specimen (in N), *l* is the distance between the support rollers (in mm), *b* is the width of the specimen (in mm), and *d* is the depth of the specimen (in mm). The mean value was then determined, and the units transformed into MPa. 

Regarding the compression test, the mean value of the compressive strength was found by dividing the maximum load carried by each specimen by its cross-sectional area.

### 2.4. Water Absorption by Capillarity Tests

The water absorption was calculated following EN 1015-18 [27] (although paraffin was not used) and EN ISO 1514 [28]. Three specimens of each mortar were used to perform the experimental program. Prior to this experiment, the samples were kept in an oven at 60 °C for 24 h and then remained for a further 24 h in a desiccator to remove any moisture that might exist. Initially, the specimens were marked with a line, at a distance of 5 mm, defining the part of the sample that should be immersed in water. This line served as a reference point to identify the height reached by the water in the sample. The water absorption coefficient (*C*) was calculated using Equation (2):

(2) $$C=0.1\times \left(m_{2}-m_{1}\right)$$

where *m*_1_ is the mass of the sample at 10 min and *m*_2_ is the mass of the sample at 90 min.

### 2.5. Contact-Angle Measurements

**Figure 3:**
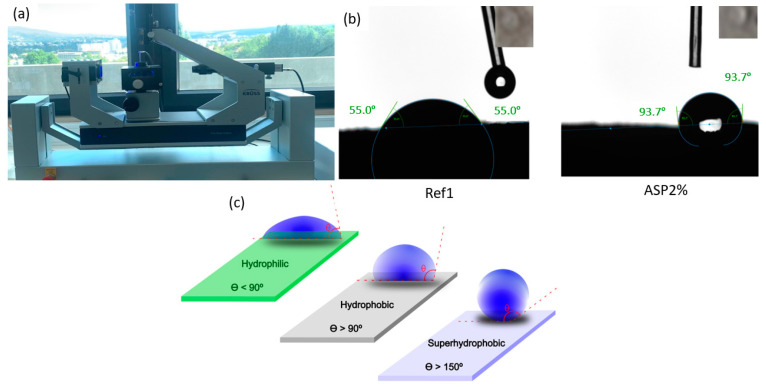
Contact-angle experimental procedure: (**a**) equipment; (**b**) frame of the contact-angle measurement; (**c**) schematic representation of the surface’s classification as a function of the water contact angle (θ).

Additionally, in order to obtain more information about the behaviors of the samples when placed in contact with water, experimental tests were carried out to evaluate the wettability of the mortar’s surfaces, allowing for the identification of their hydrophilic or hydrophobic character. The surface wettability of the samples was assessed by means of static water contact-angle measurements using the sessile drop method. Contact angles were measured in a temperature-controlled chamber at 26 ± 1 °C with ultra-pure distilled water with the volume of liquid droplets kept constant at 10 µL. A Kruss DSA25S drop-shape analyzer controlled by the software KRUSS ADVANCE 1.15.035501 was used. The digital images were acquired by a video camera using the Young–Laplace fitting. For each sample, between three to five drops of water were placed at the surface of the samples. The experiments were repeated at least three times. The classification of the surfaces as a function of the water contact-angle values is schematically represented in Figure 3. When the contact angle is <90°, the surface is called hydrophilic; for a contact angle > 90°, the surface is hydrophobic; and for a contact angle > 150°, the surface is considered superhydrophobic [29].

### 2.6. Accelerated Biological Susceptibility Evaluation

**Figure 4:**
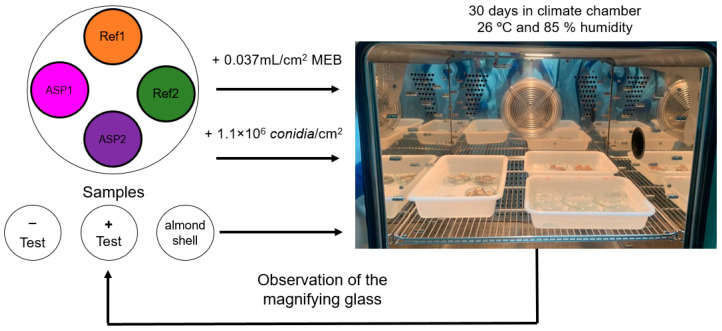
Procedure used for the fungal growth susceptibility.

For the evaluation of fungal growth, samples of Ref1, Ref2, ASP2%, and ASP4% with a circular cross-section were considered. After 28 days of curing under the ambient conditions of the laboratory, the specimens were placed in a Petri dish and sprayed with fungal spores at a concentration of 1.1 × 10^6^ *conidia*/cm^2^. Surface contamination was carried out by 6 direct sprays in the samples already placed in the Petri dish (area = 56.26 cm^2^), corresponding to 1.0 mL. The isolate MUM 19.43 (MUM—Mycotec da University of Minho) was molecularly identified as *Cladosporium halotolerans*, and its sequence was deposited on the GenBank under the accession number MN839644. The fungi had a concentration of 5.6 × 10^6^ spores/mL. The samples were placed in a climatic chamber (Memmert GmbH + Co. KG DE-91107) at 26 °C and a relative humidity (RH) of 85%. Two Petri dishes sprayed with *malt extract agar* (MEB) were used as a positive test, which were also placed in the climatic chamber. As a negative test, two Petri dishes with *Cladosporium Halotolerans* and two with MEB were also considered as a reference. The climate-chamber experiment lasted 30 days. The hygrothermal conditions and duration of the experiment are representative of a biological susceptibility acceleration (Figure 4), whose procedure has already been followed in previous studies [30,31].

## 3. Results and Discussion

### 3.1. Flexural and Compressive Strength

**Figure 5:**
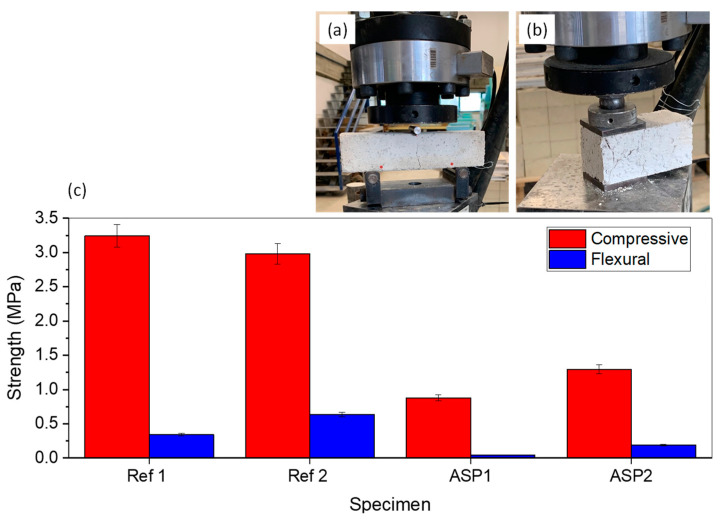
Compressive and flexural results of the lime mortars: failure mode for (**a**) flexural and (**b**) for compressive tests; (**c**) values of strength.

It may be inferred from Figure 5 that the mechanical strength of the mixtures decreased upon introduction of 2% and 4% of *ASP*. Lower values of mechanical strength were observed for the compression tests (red bars) than for the flexural ones (blue bars). However, higher values of flexural and compressive strength were achieved for ASP4% than for ASP2%. This finding may lead to the conclusion that a mechanical property’s improvement was achieved when microparticles of *ASP* were submitted to the dispersion techniques and incorporated into the lime-mortar mixtures, in contrast to what is expected when an increase of non-dispersed *ASP* is included in the mixture. 

In the present case, adding 4% of *ASP* to the mixture composition led to an increase of 13% in compressive strength when compared to ASP2% (Figure 5, red bars). In ASP2% and ASP4%, there was a decrease of 73 and 60%, respectively, in the compressive strength with respect to Ref1. Regarding Ref2, the decrease was 70% (ASP2%) and 57% (ASP4%). Despite leading to a large reduction in compression strength when compared to the control mortar, ASP2%, and ASP4% meet the standard requirements defined in NP EN 998-I [30]. While Ref1 and Ref2 mortars can be classified as CSII, ASP2%, and ASP4% mortars present the CSI classification.

As far as the flexural strength is concerned (Figure 5, blue bars), as noted above, the values obtained were much lower than those observed for compression. However, the addition of 4% of *ASP* also led to an increase in the flexural strength value when compared to ASP2%, demonstrating the advantage of incorporating a higher percentage of powder. In ASP2% and ASP4%, there were decreased values of 88% and 44%, respectively, with respect to the reference mortar Ref1. Concerning Ref2, there was a decrease of 94% (ASP2%) and 70% (ASP4%).

Based on the present findings, an increase in the percentage of *ASP* leads to the reduction of the mechanical properties compared with conventional mortars. However, it is noteworthy that, with 4% of *ASP*, these properties had an inversion with respect to ASP2% that can be justified by an enhanced rearrangement of the composite structure. This improvement in the mechanical performance, which is visible at both the flexural and compressive strength, can be related to the particle-dispersion technique used to prepare *ASP* microparticles before being introduced into the mixture, which, as previously mentioned, may lead to the material properties’ improvement. These results may indicate that preparing *ASP* microparticles through particle-dispersion techniques can counteract the disadvantage resulting from an increase in powder content.

Despite the decrease in mechanical strength, these mortars can be promising for interior applications, especially in the rehabilitation of heritage buildings, characterized by mortars with low mechanical properties, or in new constructions, as finishing coatings. 

### 3.2. Water Absorption by Capillarity

**Figure 6:**
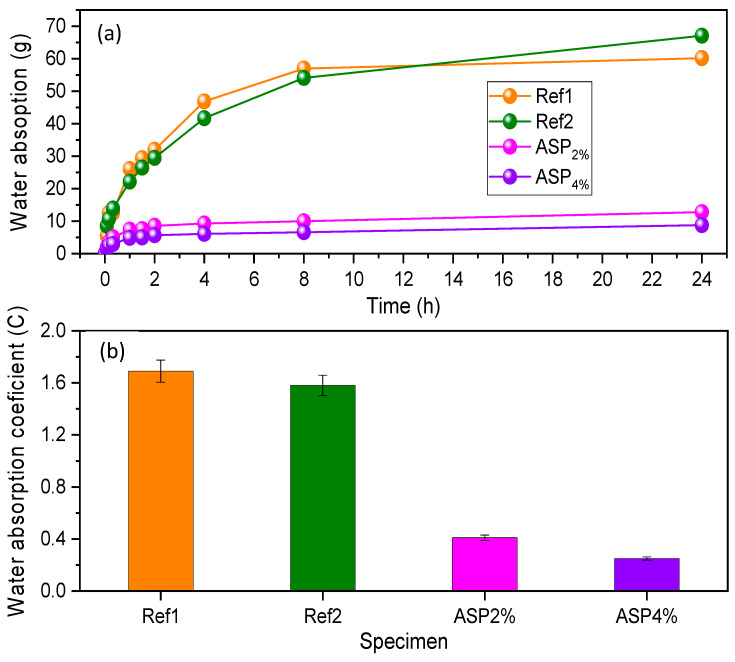
(**a**) Water absorption by capillarity and (**b**) water-absorption coefficient (*C*) of the lime mortars.

The results of the water absorption by capillarity test following EN 1015-18 [27] and EN ISO 1514 [28] are depicted in Figure 6a. It is possible to observe that Ref1 was the sample that absorbed the greatest amount of water. ASP2% and ASP4% presented a slight variation in weight, demonstrating that they both absorb a small amount of water. ASP4% absorbed less than ASP2%, a result that may be related to the increase of *ASP* content in the mixture. These results can be originated by the use of *ASP* at the microscale and the application of the particle-dispersion technique, which are in accordance with the mechanical results. There is a significant difference between Ref1, which absorbs 13% of its weight in water after 24 h, and ASP2% and ASP4% samples, which absorbed only 3% and 2%, respectively.

The *C* values estimated for the samples are shown in Figure 6b. Although lime mortars are preferably used inside buildings, it was found that ASP4% complies with the EN 998-1 [32] standard regarding outdoor applications, being classified as category W1 where *C* ≤ 0.4 kg/m^2^.min^0.5^, whereas ASP2% with *C* = 0.41 kg/m^2^.min^0.5^ is at the rank limit. Figure 6b allows for inferring that ASP4% and ASP2% absorb eight and five times less water by capillarity than Ref1, respectively. 

This characteristic exhibited by ASP2% and ASP4% is of high relevance because, if the presence of water in a material is minimized, it means that the development of fungi is hampered. Moreover, it is also possible to observe in Figure 6b that ASP2% presented a decrease in the *C* value of 80% when compared to Ref1, whereas for ASP4%, the decrease is about 88%. These results pointed out that the addition of 4% of *ASP* decreased the capacity of the sample to absorb water by capillarity, which can be an indicator that fungal growth conditions are also reduced. It also suggests that this property can be improved when microparticles of *ASP* subjected to dispersion are added to the mixture composition.

### 3.3. Wettability Studies

**Figure 7:**
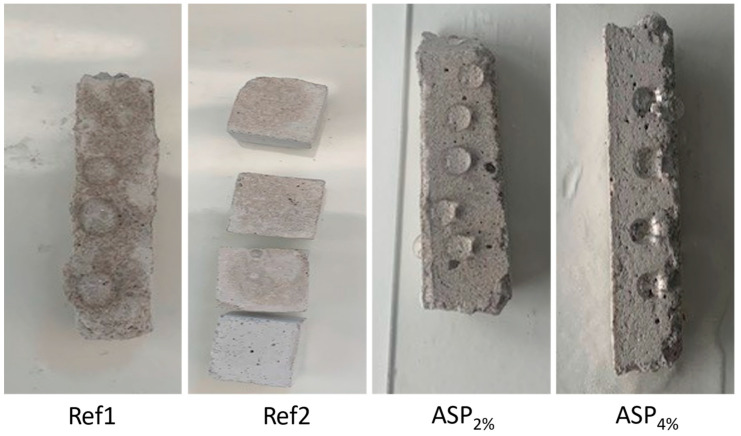
Water drops sitting along the surfaces of the lime mortars.

The results of the water contact-angle measurements carried out for the mortars under study, represented in Figure 7, provide information about the mortars’ surface wettability. Given the rather irregular surface that characterizes the mortars, 2–4 series of 15 measurements were performed. The three series of tests performed for Ref1 led to contact angles of 64.11 ± 0.55°, 77.45 ± 4.52° and 69.68 ± 8.13°, revealing hydrophilic behavior. For Ref2, the two series of measurements done led to contact angles of 63.6 ± 29.24° and 58.06 ± 14.65°, with the maximum and minimum contact-angle values being 82.69° and 16.14°, respectively. These data indicate that Ref2 has a more heterogeneous surface than Ref1 in terms of wettability. For ASP2%, the contact angles obtained for the three series of measurements carried out were 93.80 ± 2.00°, 64.59 ± 0.95° and 93.85 ± 3.00°. Finally, for ASP4%, the same series of measurements yielded contact angles of 69.53 ± 1.29° and 63.71 ± 3.93°. The results led to the conclusion that, globally, the incorporation of 2% of ASP induced an enhancement in the contact-angle value with respect to Ref1, thus decreasing the hydrophilic behavior. However, the addition of 4% of *ASP* led to a contact-angle reduction and therefore to an increase in wettability. The discussion of the above findings is not straightforward. Factors, such as dopant concentration and dopant distribution, are expected to exert key roles. These results may also be related to the heterogeneity that characterizes the mortars’ surfaces. The increase of porosity, when 4% of *ASP* is added to the mixture, results in an increase in voids, namely at the surface, allowing the drop percolation and, consequently, the reduction of the contact angle.

### 3.4. Accelerated Biological Susceptibility Evaluation

**Figure 8:**
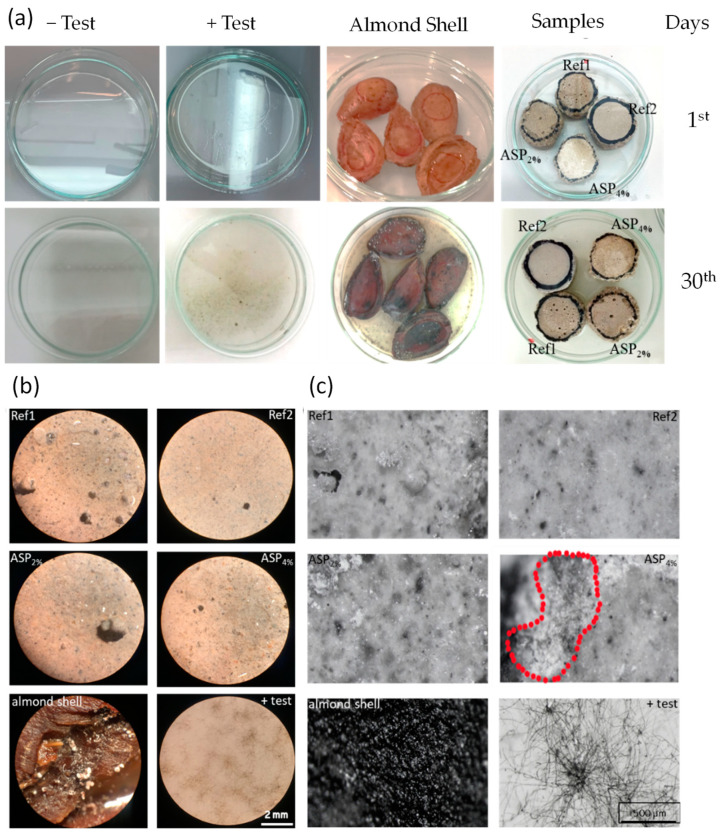
Fungal growth of the lime mortars monitored (**a**) on the 1st day and on the 30th day, and after 30 days using a (**b**) 2× and (**c**) 6.3× magnifying glass. Red dashed identifies small signs of fungal growth.

The results of the fungal susceptibility test performed over a period of 30 days, reproduced in Figure 8a, demonstrate that fungal growth is practically nonexistent in all the mortar samples. As referred to above, accelerated growth was guaranteed during a period of 30 days over which the samples were subject to controlled conditions in a climate chamber, where the procedure is similar to polymeric materials testing [33]. Figure 8b shows the samples through a magnifying glass after 30 days (NIKON SMZ800). Three visualizations were performed at different locations of each sample with 2× (Figure 8b) and 6.3× (Figure 8c) magnifications. In the analyzed samples, difficulties in the growth of fungi were also confirmed. However, the analysis of the samples with 6.3× magnification (Figure 8b) revealed some susceptibility to fungal development in the ASP4%. These results may be related to the fact that samples with 4% *ASP* presented a more porous and wettable surface when compared to the other samples. In contrast, no fungal growth was detected in the ASP2% mortar, a result that is in perfect agreement with its less hydrophilic behavior.

Thus, the obtained results revealed that adding natural compounds to lime-mortar mixtures does not increase the biological susceptibility of the composite. In fact, research work developed in this field stated that antifungal properties can be improved when these compounds are introduced into building materials. In addition, if they are incorporated at the micro or nanoscale, this contribution can significantly increase due to quantum effects [6].

## 4. Conclusions

In the present work, the potential utilization of *ASP* in the production of hydraulic lime mortars was analyzed. This almond-shell residue was added to the mortar’s formulation as microparticles, previously subjected to particle-dispersion techniques, and its influence on the antifungal susceptibility of mortars was assessed. Mechanical properties, water absorption, and wettability were also evaluated. *ASP* was added to the mixtures in the percentages of 2% and 4% in relation to lime content. A conventional mortar and a mortar containing n-TiO_2_ with recognized antifungal properties were used for comparison. The obtained results lead to the following conclusions:

- A reduction in the mortars’ mechanical properties is verified when *ASP* is added to the mixture. However, an increase in compressive and flexural strength is observed for a higher percentage of *ASP*. The introduction of 4% in the mixture led to an increase in compressive strength of 13% compared to ASP2%, which may be due to the powder’s particle size and its previous dispersion. Furthermore, ASP2% and ASP4% meet the standard requirements for mortars, as far as compressive strength is concerned;
- An increase in *ASP* content results in better behavior of the mortars to water absorption by capillarity. The reference mortar with no *ASP* addition absorbed the greatest amount of water, while the mixtures with 2% and 4% of *ASP* presented a little variation in weight, demonstrating that they both absorbed a small amount of water, which can be an indicator of lower susceptibility to fungi growth. Accordingly, a lower value of the water-absorption coefficient was observed, being reduced as the *ASP* content increased. These results suggest that changes may occur at the microstructure level, namely at the pore–structure matrix, since when 4% of *ASP* was added to the mixture, an improvement of the mechanical and water absorption behavior was achieved;
- The mortar with no *ASP* and the one incorporating n-TiO_2_ presented a hydrophilic character. When *ASP* was incorporated in the mortar formulation, less hydrophilic behavior only resulted for the introduction of 2% of *ASP*. The addition of 4% of *ASP* led to a contact-angle reduction and, therefore, to an increase in wettability, which may be related to the surface voids increase.

Difficulties in the growth of fungi were observed in ASP2%. This lower susceptibility to fungi development may be related to the decrease in the surface’s hydrophilic character. In addition, it is also suggested that an improvement in the physical and mechanical properties of the lime mortars can be achieved when *ASP* microparticles are previously subjected to dispersion techniques. This unprecedented work paves the way for the development of new sustainable bio-waste material solutions bearing functional surfaces with foreseen applications in the building sector.

## Figures and Tables

**Table 1:** Formulation of the mortars (kg/m^3^ of mortar produced).

Sample	Composition	NHL5	Aggregates	SP	ASP	n-TiO_2_	Water
Ref1	0% *ASP*	500.00	1223.15	4.00	0.00	0.00	360.72
Ref2	2% n-TiO_2_	500.00	1219.24	1.50	0.00	10.00	360.72
ASP2%	2% *ASP*	500.00	1195.06	6.00	10.00	0.00	360.72
ASP4%	4% *ASP*	500.00	1166.96	8.00	20.00	0.00	360.72

## Data Availability

Data will be available upon request.
